# The genetic organisation of prokaryotic two-component system signalling pathways

**DOI:** 10.1186/1471-2164-11-720

**Published:** 2010-12-20

**Authors:** Robert HN Williams, David E Whitworth

**Affiliations:** 1 Institute of Biological, Environmental and Rural Sciences, Aberystwyth University, Ceredigion, SY23 3DD, UK

## Abstract

**Background:**

Two-component systems (TCSs) are modular and diverse signalling pathways, involving a stimulus-responsive transfer of phosphoryl groups from transmitter to partner receiver domains. TCS gene and domain organisation are both potentially informative regarding biological function, interaction partnerships and molecular mechanisms. However, there is currently little understanding of the relationships between domain architecture, gene organisation and TCS pathway structure.

**Results:**

Here we classify the gene and domain organisation of TCS gene loci from 1405 prokaryotic replicons (>40,000 TCS proteins). We find that 200 bp is the most appropriate distance cut-off for defining whether two TCS genes are functionally linked. More than 90% of all TCS gene loci encode just one or two transmitter and/or receiver domains, however numerous other geometries exist, often with large numbers of encoded TCS domains. Such information provides insights into the distribution of TCS domains between genes, and within genes. As expected, the organisation of TCS genes and domains is affected by phylogeny, and plasmid-encoded TCS exhibit differences in organisation from their chromosomally-encoded counterparts.

**Conclusions:**

We provide here an overview of the genomic and genetic organisation of TCS domains, as a resource for further research. We also propose novel metrics that build upon TCS gene/domain organisation data and allow comparisons between genomic complements of TCSs. In particular, '*percentage orphaned TCS genes*' (or 'Dissemination') and '*percentage of complex loci*' (or 'Sophistication') appear to be useful discriminators, and to reflect mechanistic aspects of TCS organisation not captured by existing metrics.

## Background

Two-component systems (TCSs) are abundant and modular signalling pathways predominantly found in prokaryotes. The simplest and most common TCSs consist of two proteins, a histidine kinase (HK), and a response regulator (RR). HKs are typically sensory proteins that contain a conserved transmitter 'domain' (T). Transmitter 'domains' are composed of an HATPase domain wherein resides kinase activity, and a His-containing 'phosphotransfer' domain (abbreviated to P herein, typically a HisKA or Hpt domain). It should be noted that as they are a combination of two domains, the functional transmitter unit is not strictly a domain itself. However 'transmitter domain' is a term used commonly in the literature and we adopt that convention here also, as the transmitter represents a fundamental functional unit for both mechanistic and bioinformatic considerations. Upon stimulus perception the transmitter domain autophosphorylates on a conserved histidine residue within the phosphotransfer sub-domain. The phosphoryl group is than transferred onto a conserved aspartate residue in the receiver domain (R) of the partner RR. Phosphorylation of the RR causes a switch in its behaviour (often mediated by a separate 'output' domain) which leads to a cellular response to the initial stimulus. For an up-to-date series of reviews of TCS function, see Bourret and Silversmith [1] and associated articles.

Most prokaryotes possess multiple TCSs, for example *Escherichia coli *possesses 62 TCS genes, while *Bacillus subtilis *has 70 [2,3]. As all HKs and RRs are homologous, there exists the potential for any HK to signal to any RR. In reality this does not seem to happen, with most HK-RR interactions being highly specific [4-7], however identifying TCS protein partnerships from genome sequences (and thus delineating signalling pathways) remains a non-trivial task, despite significant computational advances in this area [8-10].

Luckily for researchers, in many cases the genes encoding a HK-RR pair are found adjacent in the genome, usually within a single operon (and often translationally-coupled). This arrangement allows for the coordinated expression of the two TCS genes and can provide robustness to the signalling pathway [11]. When TCS function requires additional proteins, the cognate genes are also sometimes found in an operon with the TCS genes. For example, chemotaxis operons usually contain several genes that are either required for, or modulate signalling by the Che TCS, in addition to the Che TCS genes themselves [11].

Thus contextual information regarding TCS gene organisation can be very important, providing clues regarding signalling pathway structure (RR-HK partnerships), and TCS function. However, straightforward analysis is confounded because of the expandable and modular nature of TCSs. A common example of an expanded TCS is the phosphorelay, wherein phosphoryl groups are successively transferred between His-Asp-His-Asp amino acid residues. These residues are found in successive T-R-P-R domains, which are typically encoded by two or more proteins (eg. T-R-P and R, as found in TorSR, ArcBA, and EvgSA of *E. coli *[3], or T-R-P-R, as in the sporulation phosphorelay of *B. subtilis *[12]). The second phosphorylatable histidine residue of a phosphorelay can be found in proteins that contain solely an isolated P domain, and such proteins are referred to here as phosphotransfer proteins (PP). Examples are also commonplace where 'accessory' receiver domains do not regulate effector activity directly, but rather modulate signal transduction (see for example [13-15]). Thus TCS pathway structure is often more elaborate than the basic two-component paradigm, and in such cases pathway structure cannot usually be inferred from TCS gene structure.

A further complication regards the genetic organisation of a TCS. While many TCSs are encoded as a pair of genes, often the two genes have apparently fused, encoding a 'hybrid kinase' which contains both R and T domains [16]. More complex domain architectures within TCS proteins are also common and indeed some TCS loci can be exceedingly complex. For instance, in the genome of *Myxococcus xanthus*, there are four TCS gene loci that each encode contiguous proteins containing at least two T/P domains and at least two R domains [17,18]. In such cases correlating genetic/domain organisation with signalling pathway structure often requires a *tour de force *in molecular analysis [14,19,20].

Clearly it does not necessarily make sense to describe TCSs purely in terms of the numbers of genes involved, as each gene may contain one or more TCS domains. Similarly only describing the domain organisation of a TCS and ignoring its genetic structure impoverishes understanding of that TCS. Ideally descriptions of TCSs would encompass both gene and domain organisation, and this largely happens in the published literature. However there is still no systematic nomenclature for describing TCSs [1], let alone a common categorisation system. This has led to differing classification criteria in public resources such as MiST [21,22], P2CS [23,24], and Michael Galperin's census of prokaryotic RRs [25,26], and diverse standards of description in annotated genome sequences [26]. This diversity of TCS descriptors employed, necessarily confounds inter-genome and inter-database analyses.

Ideally a categorisation scheme could be devised, which would encompass genetic and/or domain organisation as appropriate. Such categorisation would also facilitate the creation of a series of metrics allowing for meaningful inter-genomic comparisons [27,28]. At the moment the most commonly used metric for TCS genes is the straightforward quantity, the 'number of TCS genes per organism', but more advanced metrics such as IQ and extro/introvertedness have been proposed [29,30], based respectively on the number of signal transduction genes, and the proportion of sensory proteins containing transmembrane helices (a large component of both metrics being TCS genes). TCS genome metrics might valuably capture information regarding location and organisation, but would ideally do so at both gene and domain levels. New metrics could also provide mechanistic insights, as TCS gene/domain organisation appears intimately linked to mechanism (reviewed by Whitworth and Cock, [31]).

In this work we investigate the genetic and domain organisation of the TCS genes from completely sequenced genomes, and use the resulting information to develop a categorisation scheme for TCS genes. We also propose and evaluate a set of possibilities for new genome metrics, which capture diverse aspects of TCS organisation.

## Methods

### Genomic organisation of TCS genes

Data including the identity, features and replicon position of all the TCS genes from complete prokaryotic genomes (44,008 genes) were obtained from the original release of P2CS [23,24]. The location of each TCS gene was given as start and stop nucleotide positions, and the strand (+ or -) on which the gene was encoded. This allowed the trivial calculation of the distance between consecutive TCS genes (upstream and downstream, in nucleotides), and whether any two consecutive TCS genes were encoded on the same strand (*cis*) or not (*trans*). For the analyses presented here, TCS genes classified by P2CS as 'incomplete HKs' were treated alongside other HKs, while 'mispredicted TCS proteins' [24] were excluded from analysis. TCS genes were thus either HKs (including hybrid kinases), RRs, or PPs in this work (Additional File 1).

### Domain Architecture of TCS proteins

The domain architecture of each TCS protein was also obtained from the P2CS database. A list of phosphotransfer signalling domains was manually compiled and used to define phosphotransfer signalling domains within each TCS protein - other domains were ignored. TCS domains were divided into three sets: HATPase (h), receiver (R) and phosphotransfer (P). A transmitter domain (T) was defined as an HATPase domain, with an adjacent phosphotransfer domain (Ph). Isolated h domains were excluded from further analysis, including 134 genes which contained just a single h domain. All analyses were performed using custom Biopython scripts [32] and the resulting dataset was manually curated (Additional File 2).

## Results

### The relative location of TCS genes

Initially, the positions of each TCS gene within all genomes were considered, and the relative locations of each TCS gene were defined (see *Materials and Methods*). Within each replicon, the distances between consecutive TCS genes were determined and whether each pair of consecutive TCS genes was encoded in *cis *(on the same strand), or in *trans *(on opposite strands and thus either convergent or divergent).

**Figure 1:**
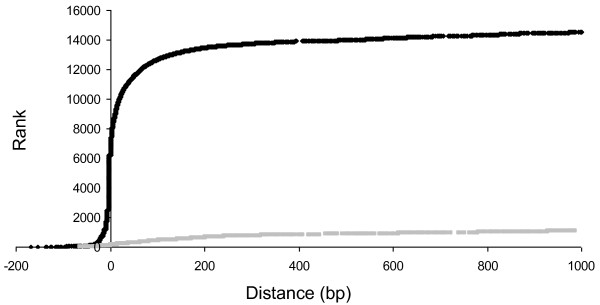
**Rank order of inter-gene distances, as a function of inter-gene distance (bp). **For *cis*-intervals (black line) there are two pseudo-linear regimes, one when distance is around 0 bp, and the other when distance >200 bp. For *trans*-intervals (grey line) there is only one linear regime, which also starts at ~200 bp.

TCS inter-gene distances were ranked in order of increasing distance, and plotted against inter-gene distance (Figure 1). The resulting curve for *cis*-intervals showed two pseudo-linear regimes. Large numbers of distances were ~0 bp (~6000, 20.3%), while for distances larger than ~200 bp, distances increased linearly with rank. The plot for *trans*-intervals only exhibited the linear regime of increasing rank with increasing distance. This regime started at a similar distance (~200 bp) to that of the *cis*-intervals, and with a similar gradient (Figure 1). Across all gene separations, the number of *cis-*intervals (29,556) was nearly double that of *trans*-intervals (13,086), with the *cis*:*trans *ratio increasing further at smaller distances (>15:1 when considering distances <200 bp). This is easily explained, as many TCS genes are found encoded in tandem gene pairs [16,33]. Additionally, around a quarter of *cis*-intervals (22.6%) were less than 0 bp, suggesting those gene pairs were overlapping - another well-documented phenomenon in TCS genes [34,28], and in prokaryotic genomes more generally [35-37].

#### Juxtapositioning of TCS genes - defining a cut-off

For both *cis*-intervals and *trans*-intervals, at larger distances there appears to be a linear relationship between rank and distance (Figure 1), implying that in this regime there is an even (random) relationship between the relative position of TCS genes. However at smaller distances, the number of TCS genes increased exponentially with decreasing distance, implying non-random juxtapositioning of TCS genes. The end of the pseudo-linear regime lies at distances of ~200 bp for both types of intervals, implying that if two TCS genes are less than 200 bp apart, then they function together, whereas if further than 200 bp apart, then their juxtaposition will normally have occurred by chance. 200 bp therefore seems an appropriate cut-off value to define whether TCS genes are co-located for functional reasons, or purely by chance. Extrapolation of the linear 'random' regime implies that imposing a 200 bp cut-off would result in a false positive rate of <2.5% for an average genome - i.e. two TCS genes that are found by chance within 200 bp of each other but are not signalling partners, would be misinterpreted as paired in under 2.5% of cases.

An orphan is defined as a TCS gene that is not adjacent to another TCS gene. By undertaking a count of orphan TCS genes as a function of cut-off distance, it was found that the number of apparent orphans decayed linearly with distance if a cut-off of >200 bp was used, however exponentially increasing numbers of orphans were identified as the cut-off distance was decreased below 200 bp.(data not shown). Extrapolation from large cut-off behaviour, implies that using a 200 bp cut-off might reduce the apparent size of clusters, but only in <2.5% of cases.

### TCS gene clusters and foci

TCS genes from 1406 replicons (44,008 genes) were grouped into gene clusters, with a cluster defined as a set of contiguous TCS genes, none of which are separated by >200 bp. Cluster size (number of genes) was recorded, with orphans having a cluster size of 1. The number and order of all TCS genes found in each cluster was also captured. Across all replicons tested, there were 16,596 orphans, 24,380 paired TCS genes, 2,268 genes in clusters of size three (triads), and 764 genes in clusters of size four (tetrads) - see Additional File 1.

Functionally inter-dependent TCS proteins are found to be encoded *in trans *very rarely, and the juxtapositioning of TCS genes *in trans *is far less frequent than juxtapositioning *in cis *(Figure 1). The effect of ignoring the adjacency of TCS genes *in trans *was investigated by defining a TCS gene 'focus' as a series of TCS genes, none of which are separated by >200 bp, and all of which are encoded on the same strand (all *cis*). As can be seen in Table 1, the numbers of genes encoded in pairs changed little when considering foci rather than clusters (1.4% reduction), although with a slight increase in the number of orphans (6.0%), and a decrease in the number of triads and tetrads (Table 1), as would be expected. The overall ratio of genes encoded as orphans, pairs, triads or tetrads varied little whether considering clusters or foci (Table 1). All following analyses were therefore undertaken on TCS gene foci rather than gene clusters.

**Table 1:** Distribution of TCS genes between different gene organisations.

		Orphans	Paired Genes	Triad Genes	Tetrad Genes
Clusters:	Numbers	16596	24380	2268	764
	Percentage	37.7	55.4	5.2	1.7
	Ratio	0.68	1.0	0.09	0.03
Foci:	Numbers	17590	24042	1788	588
	Percentage	40.0	54.6	4.1	1.3
	Ratio	0.73	1.0	0.07	0.02

Genes are categorised into clusters of foci of size 1 (orphans), 2 (pairs), 3 (triads) and 4 (tetrads). See text for details. Ratios were calculated relative to the number of genes in pairs (the greatest values).

### TCS gene organisation

The domain composition of each TCS gene focus was then elucidated as described in *Materials and Methods*. After removal of genes encoding HATPase domains without phosphotransfer domains and manual curation of genes apparently mis-classified by P2CS, a dataset was obtained containing 43,426 genes, distributed over 29,962 gene foci (Additional File 2). Genes were classified as HKs (including hybrid kinases), RRs and PPs.

#### HK and RR genes are 'evenly' distributed

Across all genomes interrogated, orphan TCS genes (17,378) were almost as likely to be HKs as RRs (45.2% and 51.0% respectively when considering foci), see Table 2, with the remainder being PPs (3.8%). Of TCS gene pair foci (11850), the vast majority (91.8%) contained a single HK and a single RR. 94.0% of triads (553 of 588) contained both HK and RR genes, with a slight preponderance (55.5%) towards those containing two RR and one HK gene. As TCS genes in our analysis were classified as either HKs, RRs or PPs, 81 different tetrad geometries/organisations were possible (3^4^). However two of the possible geometries accounted for 58.9% of all observed tetrads. Those two geometries each contained two RR and two HK genes, in an alternating order (i.e. HK,RR,HK,RR and RR,HK,RR,HK). When all tetrads comprising two HK and two RR genes were considered, irrespective of order, they accounted for 71.2% of all tetrads, with no other single tetrad geometry accounting for more than 6.2% of the total (146). It therefore seems that in many ways (relative numbers, organisation, ordering), there is a 'constancy' regarding HK and RR gene distribution - i.e. for every RR there is a HK (and *vice-versa*) - thus at the genetic level the basic unit of a TCS is apparently a HK-RR gene pair. This of course correlates with the mode of action of the typical TCS - the direct transfer of phosphoryl groups between paired HK and RR proteins.

**Table 2:** Total numbers of HK, RR and PP genes as a function of the number of TCS genes at a focus.

		HK	RR	PP	# of foci
# Genes	1	7854	8858	666	17378
in focus	2	11395	11999	306	11850
	3	791	936	37	588
	4	314	265	5	146
	Total	20354	22058	1014	

#### Phosphotransfer protein genes

Consistent with this observation, across all genomes, RRs and HKs exhibit a remarkably similar distribution of gene organisations (39.4 ± 1.1% found as orphans, 55.2 ± 1.1% in pairs, and 5.4 ± 0.0% in triads and tetrads). However, the distribution was found to be very different for PPs, with nearly two-thirds (65.6%) found as orphans, and only 30.2% in gene pairs (Table 2). Thus PPs appear to exhibit less 'constancy' than HKs or RRs, being more likely to be found as orphans, and with a reduced frequency of being found in a partnership. When they were found in a pair, the partner gene was a RR in the majority of cases (85.0%). It could be argued that at the genetic level, if there is a functional unit involving PP genes, it is a RR-PP unit. This proposition also seems obvious, as PP genes are predominantly found within HK-RR-PP-RR phosphorelays, which are typical HK-RR TCSs expanded by the inclusion of an RR-PP unit.

The presence of PP genes was also restricted to a small set of possible triad and tetrad geometries. 37 PP genes were found in triads, and of those, 30 (81.1%) were found alongside one RR and one HK gene. Of the five PP genes found in tetrads, only one was found in a HK > RR > RR > PP > focus (5' to 3') reminiscent of a phosphorelay, while the remaining four were found in foci with the geometry HK > RR > PP > HK >, suggesting the encoding of a signalling pathway structure more complex than that of a standard phosphorelay.

#### Plasmid-encoded TCS genes

As plasmids are relatively mobile/dispensible genetic elements, the properties of plasmid-encoded TCS genes (797 genes) were compared to those of the whole genomic dataset (see Additional File 1). Most plasmids (75.4%) encoded no TCS genes, however on average there were 1.38 TCS genes per plasmid across all genomes. Twenty plasmids encoded at least 10 TCS genes each, and 18 of those came from Alphaproteobacterial or Betaproteobacterial hosts - groups composed largely of symbiotes and parasites of metazoans.

The chi-squared test was employed to compare the observed numbers of plasmid-encoded orphans, pairs, triads and tetrads, with those expected from whole dataset distributions. It was found that the distribution of plasmid encoded TCS genes was significantly different from that of the whole genomic dataset (*p *< 0.0001). Specifically, there were more orphans than expected (367 rather than 319), and fewer paired genes (366 rather than the 435 expected), with little difference for triads and tetrads (48 and 16 respectively observed, 32 and 19 expected). However despite these differences, the composition of the 'orphan' and 'paired gene' classes of plasmids were virtually identical to those of the whole genomic dataset (HKs and RRs were 46.9% and 50.7% of the plasmid orphans respectively, while paired RR and HK genes made up 90.7% of the plasmid paired gene foci). Therefore despite the relative mobility of plasmids, they tend to contain fewer paired gene systems, instead encoding a greater proportion of TCS genes as orphans.

#### Phylogenetic aspects of TCS gene organisation

127 replicons encoded more than 100 TCS genes each (Additional File 1). Of those replicons, tetrads accounted for at most 9.0% of the TCS complement (*Rhodopseudomonas palustris*, BisA53), while nearly half (58) of the replicons had no tetrads. Tetrads were particularly enriched in the Alpha-, Beta-, and Delta- proteobacteria (16 of the top 20 replicons were from those clades). Triads were found to comprise up to 15.2% of one replicon (*Geobacter lovleyi *SZ), and again triads were particularly common in the chromosomes of the Alpha-, Beta-, and Delta- proteobacteria (14 of the top 20). Only 15 of the 127 replicons had no triads, 11 of which were from Firmicutes. Conversely, paired genes were most common in the Firmicutes (13 of the top 20), with the percentage of TCS genes found in pairs ranging from 82.3% (*Clostridium phytofermentans *ISDg) to 11..5% (*Xanthomonas oryzae pv. oryzae *PXO99A). A similar range was found in the percentage of TCS genes found as orphans, which varied reciprocally with the percentage of paired genes. The percentage of orphan TCS genes ranged from 85.6% (*Xanthomonas oryzae pv. oryzae *PXO99A) to 14.5% (*Clostridium phytofermentans ISDg*). As expected, it seems there is a significant phylogenetic dimension to the organisation of TCS genes.

### TCS domain organisation

As noted above, the distribution and numbers of RR and HK genes in replicons are very similar. However across all prokaryotic genomes, there are around four receiver domains for every three transmitter domains [28]. The reason for this apparent discrepancy is that a large proportion of HK genes (22.8%) encode proteins with a receiver domain(s) in addition to transmitter domains - such proteins are usually described as hybrid kinases. Therefore to truly understand/explain the organisation of TCS genes, it is necessary to take into account the various combinations of domains found within TCS genes.

Phosphotransfer signalling domains within each TCS gene were identified and classified as either transmitter (T), receiver (R), or phosphotransfer domains (P), see *Materials and Methods *for details. This allowed each TCS gene and focus to be defined as a string of 'R', 'T', 'P' and '-'s, where '-' denotes a junction between genes. Inspection of the resulting manually-curated dataset (Additional file 2) confirmed that receiver domains are significantly more numerous than transmitter domains, both of which outnumber phosphotransfer domain by around an order of magnitude (27,839 R, 20,408 T and 2,197 P domains).

#### Domain organisations within TCS proteins

Initially the nature of domain architectures and their frequency within the TCS gene population was investigated (Tables 3, 4, 5, 6 and 7). On average, TCS genes each possess 1.16 TCS domains (Table 5). The majority (86.2%) of TCS genes encoded single TCS domains, with receiver domain-only proteins (21,614) more frequent than transmitter domain-only proteins (15,691) and with a relatively small number of phosphotransfer proteins (1,012). A small proportion (1.4%) of RRs possessed multiple receiver domains (Table 6), generally two (301 examples), but three in 14 cases. Non-hybrid HKs rarely possessed multiple transmitter domains (two of 15704 proteins), however the proportion of proteins with two transmitter domains was significantly enriched in hybrid kinases (51 of 4650). No proteins were found to contain more than two transmitter domains. Hybrid kinases were also enriched for multiple receiver domains (Table 6), with 1113 of 4650 hybrid kinases possessing 2 or more receiver domains. Of HKs possessing a single transmitter domain, the majority encoded no receiver domains (77.3%), however virtually all HKs with two transmitters possessed receiver domains (96.2%), presumably to somehow modulate/integrate signalling between the two transmitters.

**Table 3:** The number of genes containing R, T and P domains as a function of the number of domains within the gene.

	Genes with ≥1:	T	R	P	# of genes
# Domains	1	15691	21614	1012	38317
in gene	2	3202	3591	137	3605
	3	1105	1135	707	1136
	4	331	343	244	343
	5+	25	25	17	25
	Total	20354	26708	2117	

**Table 4:** The number of foci encoding R, T and P domains as a function of the number of domains at that focus.

	Foci with ≥1:	T	R	P	# of foci
# Domains	1	4660	8556	664	13880
in focus	2	12506	13353	367	13420
	3	1443	1521	525	1525
	4	746	758	348	759
	5+	376	378	197	378
	Total	19731	24566	2101	

**Table 5:** The number of TCS domains per TCS protein.

TCS domains	1	2	3	4	5	6	7+	Total
# Proteins	38317	3605	1136	343	20	2	3	43426

**Table 6:** Receiver (R) and transmitter (T) domains found in TCS proteins.

Domains	0R	1R	2R	3R	4R	5R	6R	Total
0T	1014	21743	301	14	0	0	0	23072
1T	15702	3919	588	88	4	0	0	20301
2T	2	33	13	4	0	0	1	53
Total	16718	25695	902	106	4	0	1	43426

**Table 7:** The number of phosphotransfer domains (P) in TCS proteins, and whether the proteins also contains transmitter and/or receiver domains.

Domains	0P	1P	2P	3P	4P	5P	6P	Total
≥1 T	19416	887	37	10	2	0	2	20354
≥1 R	25617	1035	42	10	2	0	2	26708
≥1 T AND ≥1 R	3723	877	36	10	2	0	2	4650
≥1 T OR ≥1 R	41309	1046	43	10	2	0	2	42412
P only	0	1012	2	0	0	0	0	1014

88.7% of two-domain proteins contained paired receiver and transmitter domains, with a further 7.7% comprising two receiver domains. Two organisations were also particularly common in proteins comprising three TCS domains - 679 proteins (59.8%) had one of each domain type (TPR), while 392 proteins (34.5%) possessed two receiver and single transmitter domains. Three combinations of domains (independent of order) accounted for 93% of proteins possessing four domains - 196 proteins were TRRP (57.1%), 86 were TRRR (25.1%), while 36 were TRPP (10.5%). Proteins possessing more than four TCS domains tended to comprise multiple Hpt domains upstream of a TR unit (10 of the 20 5-domain proteins were PPPTR, both 6-domain proteins were PPPPTR and both 8-domain proteins were PPPPPPTR). The only protein encoding 9 TCS domains, was RRTRRPRTR, being Mmc1_3271 from *Magnetococcus sp. *MC-1, a magnetotactic Proteobacterium [38].

Proteins containing a phosphotransfer domain either possessed no other TCS domains (1014 proteins), or tended to also possess *both *transmitter and receiver domains (877 proteins), presumably forming parts of phosphorelays (Tables 3 and 7). Of the 1046 P-containing proteins which also had receiver and/or transmitter domains, 1000 (95.6%) had domain complements (independent of order) of either TRRP (196), TRP (679), or PR (125), which are likely members of typical phosphorelays [39]. Intriguingly, TRP proteins were predominantly orphans, only 30.5% (207) were encoded adjacent to a single response regulator, which would be required for the focus to encode an intact phosphorelay. Conversely, 42.3% of TRRP proteins (83) were encoded next to single RR genes, suggesting that a large proportion of TRRP proteins may not encode an intact phosphorelay by themselves.

### Domain organisation of TCS gene foci

The domain architectures of entire TCS gene foci were then categorised, as described in *Materials and Methods *(see Additional File 2). Nearly half the TCS gene foci (13880, 46.3%) encoded a single TCS domain (Table 8), and of those the majority comprised a single receiver domain (8,556, 61.6%). Such proteins were nearly twice as numerous as genes encoding a single transmitter domain (4,660, 33.6%), with single phosphotransfer genes being relatively rare (664, 4.8%). A similar number of foci encoded two TCS domains (13,420). The majority of two-domain foci (10,834, 80.7%) consisted of two genes, 93.6% of which comprised a classic HK-RR pair. Of the 2,586 orphan gene, two-domain foci, 2,304 (89.1%) were hybrid kinases, thus constituting an entire TCS. Considering foci encoding more than two TCS domains, observed numbers decreased rapidly, as the number of encoded domains increased (Table 9).

**Table 8:** The relative frequencies of foci with different numbers of encoded TCS domains.

Domains	1	2	3	4	5	6	7	8+	Total
# foci	13880	13420	1525	759	241	77	43	17	29962

**Table 9:** The number of genes at a focus, as a function of the number of domains encoded at a focus.

		Genes per Focus				
	# Genes	1	2	3	4	Total
Domains	1	13880	-	-	-	13880
per	2	2586	21668	-	-	24254
focus	3	700	1072	867	-	2639
	4	192	646	489	324	1651
	5+	20	314	408	260	1002
	Total	17378	23700	1764	584	43426

#### Genes or foci, domains or genes?

Is it more sensible to consider TCS organisation at a focus level, or at a gene level? We could start with the assumption that the most important factor is the number of TCS domains at a focus, with the distribution of those domains between genes being less important. If so, we would expect that for any number of encoded domains, the proportion of genes containing T, R or P domains, would be similar to the proportion of foci containing those domains (Tables 3 and 4). This is generally the case, to within 5%, however in some cases there is a great difference between foci and genes, especially considering 1-domain foci, and foci containing phosphotransfer domains. For instance, phosphotransfer domains are found in 34.4% of 3-domain foci, but in 62.2% of 3-domain genes. Phosphotransfer domains are similarly enriched in 4-domain and 5+-domain genes. Presumably, in a three-domain protein, a phosphotransfer domain would normally be required to signal to another protein (potentially encoded by an adjacent gene) completing a phosphorelay. Conversely phosphotransfer domains within 3-domain foci might be less common because to retain functionality, they would have to interact with proteins encoded by more distant genes.

For single domain foci, the proportion containing a transmitter domain is 33.6%, however the percentage of single domain genes with a transmitter domain is 41.0%. Presumably this is because non-hybrid HKs will have a partner RR, and there is a selective pressure towards the co-location of the two genes. Conversely, the percentage of proteins encoding a receiver domain is 61.6%, while the percentage of single domain genes that contain a receiver domain is only 56.4%. This may suggest there is a pressure towards the 'orphanisation' of some receiver-domain only genes, potentially because they have multiple signalling partners.

#### Plasmid-encoded TCS foci

As described earlier, more than half of all plasmid-encoded TCS were found on just 20 plasmids, each of which encoded more than 10 TCS genes. Across all genomes R, T and P domains are found at frequencies of 55.2%, 40.5% and 4.4% respectively, however in plasmids the relative frequencies of domains is significantly different (*p *< 0.001), with 55.2% R, 36.5% T and 8.4% P domains. Thus for plasmid TCS, it seems that phosphotransfer domains are over-represented, at the expense of transmitter domains. Assessing the number of domains encoded at a focus, there were also significant differences between plasmid foci and those of the entire genomic dataset (*p *< 0.0001), with 60.0% of foci encoding a single domain (45.4% for the whole dataset), with a corresponding reduction in the number of paired-gene foci (89 observed, 132 expected).

#### Phosphorelays

In a typical phosphorelay, phosphoryl groups are successively transferred between T, R_1_, P and R_2 _domains. In an early review of phosphorelay biology, Appleby *et al.*, [39] noted that the constituent domains of some phosphorelays exhibited different patterns of covalent linkages. For instance, some phosphorelays were distributed over four proteins, while others were composed of three or even two proteins.

In our dataset there were 759 foci that consisted of four domains, dominated by four domain-combinations (307 TRPR, 262 TRTR, 136 TRRR and 38 PPTR). Of the 307 proteins containing TRRP domains, 85 were encoded as one gene, 210 were distributed over two genes, 11 were three-gene systems and only one focus consisted of four genes. Of the 210 two-gene systems, 209 were found as a TRP-R focus, while 9 of the 11 3-gene foci had a TR-P-R geometry. Thus for TRPR systems, there is a distinct bias towards two of the possible 8 geometries (T-R-P-R and TRP-R), together accounting for 95.8% of cases. Conversely for the 262 TRTR foci, every possible geometry was seen at least 12 times, with no single geometry accounting for more than 27.1% of cases (71 instances, T-R-T-R). There were also 241 foci encoding five domains in the dataset, and nearly half of these of those (105) have a TRRRP domain combination, reminiscent of a phosphorelay with an extra receiver domain. 81 (77.1%) of these were arranged in a two gene organisation TRRP-R similar to the TRP-R geometry found in 68.1% of TRRP foci.

### Metrics of TCS signalling

As can be seen above, the TCS complement of genomes can be characterised in many ways, and at several levels. An understanding of the relationships between TCS foci, genes and domains can help us to infer the functional properties of TCSs from their gene sequence and context alone. It also enables us to define features of 'normal' TCS signalling, and in doing so identify organisms/systems in which TCS signalling is abnormal and thus interesting/novel. Metrics have already been proposed that allow comparisons between organisms, based on the abundance and character of signalling genes in a genome [29,30]. These metrics correlate the number of signalling genes (including TCS) in a genome with IQ, and the proportion of sensory proteins lacking transmembrane helices with 'introvertedness'. Another metric based on the relative abundance of different families of signalling proteins (again including TCS), has been shown to be consistent within phyla, and yet capable of discrimination between closely-related organisms with differing signalling capacities [29].

In the following section we describe the rationale behind a series of potential metrics and evaluate their ability to discriminate between the TCSs of various organisms.

#### Measures of TCS genes/domain numbers

Clearly the '*number of TCS genes/domains*' is an important measure of prokaryotic adaptability. The number of TCS regulators is taken into account when calculating bacterial IQ [29] and in some organisms this will be a major component of the IQ score. Thus the number of TCS regulators, whether assessed in genes or domains, will scale with IQ (although the correlation between IQ and the number of TCS genes/domains will depend upon phylogeny).

The '*percentage of TCS genes on plasmids*' varies quite widely between organisms, from 0.0% to 56.3% (*Sinorhizobium medicae *WSM419 and *Sinorhizobium meliloti *1021 possess more than 60 plasmid-encoded TCS genes each). However 87.4% of organisms with TCS genes have none encoded on plasmids, and those with large numbers of plasmid-encoded TCS genes are predominantly Alpha- and Beta-proteobacteria.(see above). No organisms outside the Alpha- or Beta-proteobacteria contained more than 25% plasmid-encoded TCS, further limiting its use as a potential metric.

The domain/gene organisation of organisms could potentially form the basis of metrics. The '*percentage of orphan TCS genes*' and the '*percentage of paired TCS genes*' both range from 0 to 100%, and both still range over at least 67.8% even when only considering replicons with >100 TCS genes. Of those genomes, *Xanthomonas oryzae pv. oryzae *PX099A, exhibited the greatest proportion of orphaned TCS genes (85.6%, more than 10% greater than the second highest scoring organism, *Anabaena variabilis *ATCC 29413), while only 14.5% of the TCS genes of *Clostridium phytofirmans *ISDg were orphaned.

The presence of PP genes (and phosphotransfer domains) is indicative of an expanded TCS, so counts of the '*number of PP genes*' was considered as the basis of a metric. However very few genomes possess multiple PP genes - only 24 replicons encoded more than 4 PP genes (*Flavobacterium johnsoniae *UW101 possessed the most, with 14), and 52.5% of chromosomes possess no PP genes, substantively limiting it's use as a metric. Even focussing on phosphotransfer domains rather than PPs does not improve such a measure's usefulness significantly, as there are only three times more phosphotransfer domains than PP proteins across all genomes.

For similar reasons, '*average genes/domains per focus*' is of limited use as a discriminatory metric, as only 8.9% of foci have more than two domains, and only 2.4% of foci have more than two genes. Indeed as 82.5% of all TCS foci are either one-gene, one-domain foci, or two-gene, two-domain foci, measures of '*domains per focus*' correlate with the '*% of paired TCS genes*' and '*% of orphan TCS genes*' measures. For example, '*average number of genes per focus*' is negatively correlated with '*% of orphan TCS genes*' with *R*^2 ^= 0.87.

#### Measures of complexity

Potential metrics described thus far have centred around various measures of the number of domains/genes per focus. It is also possible to devise metrics based upon degrees of complexity within TCS foci. For example one might assess the number of different TCS gene organisations within a replicon, or the proportion of foci with >*n *genes. We compared four such metrics: the number of different focus organisations or '*diversity*', the number of different focus organisations comprised of >2 genes ('*diversity >2*'), the number of foci containing >2 genes ('*# >2*'), and the percentage of foci containing 2 or more genes ('*% >2*'). '*# >2*', '*diversity >2', and 'diversity*' all correlated with the '*number of TCS genes*', with *R*^2 ^values of > 0.74. This might have been expected as the larger the number of TCS genes within a genome, the greater the number of more complex focus organisations. However '*% >2*' was found to correlate only slightly with '*number of TCS genes*' (*R*^2 ^= 0.0607 across all replicons, and *R*^2 ^= 0.0922 for replicons with ≥50 TCS genes). Thus it would seem that the '*% >*2' metric captures some feature of TCS organisation distinct from the '*number of TCS genes*', or the '*number of genes/domains per focus*'. Applying the '*% >2*' measure to replicons encoding 50 or more TCS foci, scores ranged from 22.8% (*Burkholderia phymatum *STM815 chromosome 1) to 0%. A significant proportion of replicons (32.7% of replicons containing ≥50, but only 6.5% of replicons containing 100 or more TCS genes) possess no foci of >2 genes, which restricts the metric's applicability to replicons with large numbers of TCS genes.

## Discussion

Assessing the relationship between TCS gene adjacency and nucleotide distance, led to the proposal that 200 bp be used as a cut-off for the definition of functionally co-located TCS genes on the same DNA strand. The imposition of such a cut-off gives similar results to manual analysis of TCS gene structure. For example using a manual approach [33]*Bacillus subtilis *has been described as having 10 orphan TCS genes, and 60 paired genes, whereas using the automated scheme here, *B subtilis *apparently encodes 58 paired genes and 12 orphans. 200 bp is a commonly used cut-off for defining likely operons [40,41], and restricting focus membership to genes within an operon is a desirable outcome. A cut-off of 200 bp is pessimistic - it will often cause the separation of genes that would intuitively be assumed to be co-functioning, reducing the apparent size of TCS gene foci. However, a pessimistic cut-off reduces the problem of mis-classifying genes as being co-functional purely because of the relative proximity of their genes, which is more of a problem for replicons with higher densities of TCS genes. Basing classification on a distance cut-off makes the scheme presented here sensitive to mis-classification of start sites, which is still an unsolved problem in genomics, however it does avoid the problem of gene underestimation during annotation.

Other categorisation schemes have used *ad hoc *sets of rules for assessing adjacency of TCS genes [22,33]. For example, the MiST2 database assesses linkage by restricting analysis to unidirectional gene clusters (wherein no genes are >200 bp apart) within which linked TCS genes could be separated from each other by up to two other genes [22], while Whitworth and Cock [33] used an arbitrary 5,000 bp cut-off. Both or these approaches are thus far more optimistic classification schemes than the approach used here and don't have the benefit of being based on gene-distance data. Despite this, our results are similar to those of other studies. Applying the categorisation scheme to 43,427 TCS genes from the P2CS database (version 1), revealed that the vast majority of TCS genes are encoded as either orphans (40.0%) or paired genes (54.6%), while the MiST database classifies 38% of TCS genes as orphans [22]. In a recent upgrade to the P2CS database, the classification scheme defined here has been adopted, providing free access to gene organisation information for all TCS in completely sequenced prokaryotic genomes and metagenomes [42].

In addition to noting gross trends of TCS organisation, categorisation of TCS genes allowed analysis of previously unexplored aspects of TCS gene organisation from a multi-genomic perspective.

Analysis of the distribution of TCS genes between chromosomes and plasmids showed that plasmids are relatively enriched for orphan TCS genes, and that phosphotransfer domains are over-represented in plasmids at the expense of transmitter domains. The relative abundance of phosphotransfer domains implies that plasmid-encoded TCS tend towards the more complex 'expanded' types. Plasmid TCS genes also tend to be orphaned, which suggests that plasmids either tend to encode more TCS pathways with components distributed around the plasmid, or encode more TCS proteins that act in combination with, or by modulating the activity of, chromosomally-encoded TCS. Intriguingly, a recent study of *Rhodococcus equi *has suggested that a plasmid-borne orphan RR required for virulence (Orf8), is activated by a chromosomally-encoded HK (MprB), providing evidence that this latter scenario is plausible [43]. In either case, it is not clear why plasmid-encoded TCS should be at all different from chromosomally-located TCS.

Classically, phosphorelays have been described as TCSs involving phosphotransfer between four successive domains (T > R_1 _> P > R_2_), as described by Appleby *et al.*, [39]. Such phosphorelays are readily identified in our datasets. Most 4-domain TCS foci encoding T, R, R and P domains were found as either a TRPR single gene (85, 27.7%), or as a TRP-R two-gene focus (209, 67.4%). Other relatively uncommon domain architectures are also viable functional units however - for instance a TR-P-R system (YsrRST) has been recently described in *Yersinia enterocolitica*, [44]. A single domain/gene organisation is found for 77.1% (81) of the 105 5-domain foci containing TRRRP domains (TRRP,R), suggesting that these foci encode phosphorelays whose activity is somehow modulated by an extra receiver domain, a suggestion made previously [45]. An example of an experimentally characterised system showing such complexity is the CbbRRS phosphorelay of *Rhodopseudomonas palustris*, which comprises a TR hybrid kinase (CbbSR), a PR protein (CbbRR1) and a further RR (CbbRR2) [46]. Also abundant in our datasets were loci encoding two transmitter domains and multiple receiver domains. The genetic organisation of these loci (often with alternating receiver and transmitter domains) suggests they might encode phosphorelays where the function of the Hpt phosphotransfer domain of classical phosphorelays is performed by the phophotransfer domain within the second transmitter domains of the system. Alternatively such loci may just be the consequence of the coincidental juxta-positioning of two dyad TCSs. However, one such system that has recently been experimentally characterised is the *redCDEF *system of *M. xanthus *[14]. The Red pathway is encoded by a T_1_-R_1_R_2_-T_2_-R_3 _focus and the T_2 _domain has been shown to receive phosphoryl groups from the R_1 _domain (the T_2 _domain appears incapable of autophosphorylation). In addition, for the TRT-R phosphorelay TodST of *Pseudomonas putida*, the second transmitter domains has been shown to act as a phosphotransfer domain, while possessing autokinase activity [47]. It therefore seems likely that TRTR phosphorelays are nearly as commonplace as TRPR phosphorelays, although currently less-well studied experimentally.

As noted by Appleby *et al. *[39], TCSs display different covalent linkages between their TCS domains. Cock and Whitworth [16] investigated the gene/domain relationship for 'minimal' TCS foci - those encoding a single transmitter and receiver domain pair. It was found that whether the two signalling domains were found as one or two proteins was dependant on whether the TCS possessed a transmembrane sensory domain, or a DNA-binding output domain, each of which require particular sub-cellular localisation for function [16]. Presumably arguments of co-localisation will affect the distribution of domains between genes, and the distribution of genes between foci, for all TCS geometries. The complexity of addressing such a topic computationally for pathways more complex than a minimal TCS is daunting though, and must be left for the future. However exploring the relationship between domain and gene organisation has the potential to unveil mechanistic features of TCS signalling/evolution, as it has done for minimal TCS [16]. Hopefully the analysis and datasets presented here open up this possibility.

Our analysis interrogated the collection of completely sequenced prokaryotic genomes. Features of TCS gene/domain organisation are not conserved globally, with different phyla exhibiting distinct properties, for example Firmicutes tend to encode TCS as pairs and not orphans, whereas Proteobacteria tended to encode most of the TCS gene triads and tetrads. The dataset used to draw our conclusions is biased towards highly sequenced clades. The impact of such sequencing bias can be reduced by removing redundancy at an arbitrary taxonomic level (see for example [26]), but ideally the analysis presented here would be performed individually for separate taxa, as depth of sequencing allows. Unfortunately for most taxa there are currently not sufficient complete genome sequences available to undertake such analysis at even the order level, nor sufficient data on lifestyle to allow correlations with life history traits [28]. Nevertheless, assessing the properties of TCS genes across all genomes is useful, as it provides a context for the analysis of individual organisms, groups of organisms, and subsets of TCS proteins, for instance CheA homologues (HKs with a Hpt rather than HisKA phosphoacceptor domain), or an individual family of RRs.

Finally we investigated possible metrics of TCS gene organisation. Useful metrics are able to distinguish between a wide variety of organisms, and provide a measure of an organismal feature that isn't captured by other metrics. Some metrics capture phenotypic properties, for example extrovertedness and IQ/adaptability which reflect lifestyle and ecology [29], while others reflect mechanistic features of a system/organism, such as the relative numbers of one-component and two-component systems [48], providing insights into evolutionary pressures and molecular mechanisms. In addition to providing such insights, metrics also allow rigorous rather than intuitive comparison between organisms.

Here we propose two metrics that capture information regarding the genetic organisation of TCSs - the '*percentage of orphan TCS genes*', and the '*percentage of TCS genes in foci of >2 genes*'. For ease of reference we have assigned these metrics the anthropomorphic names 'Dissemination' and 'Sophistication' respectively. The orders of organisms ranked by the two metrics are not significantly correlated, nor do they correlate with ranking by the number of TCS genes (a correlate of IQ). Care needs to be taken when describing the degree of Dissemination and Sophistication of organisms that encode few TCS genes, as the scores are percentages, and as the number of TCS genes per replicon drops, the proportion of replicons with no Sophisticated foci increases. Dissemination is strongly and negatively correlated with the percentage of paired TCS genes (*R*^2 ^= 0.92), and would also be expected to scale with the related measures of percentage paired/orphan transmitter/receiver domains.

The distribution of Discrimination scores has a mean of 44.1% (± 28,1%) when considering all replicons that encode TCS genes (42.3 ± 13.4% for replicons of ≥100 TCS genes), whereas Sophistication scores average 3.6 ± 7.4% (7.4 ± 4.8% when considering replicons with ≥100 TCS genes).

There appears to be little phylogenetic bias towards high Dissemination scores - restricting analysis to organisms with >100 TCS genes, the twenty organisms with the highest Dissemination scores were 2 Spirochaetes, 11 Proteobacteria, 4 Cyanobacteria, 1 member of the Euryarchaeota, 1 Planctomycete and 1 member of the Chloroflexi. The twenty replicons with highest Sophistication scores included 1 Spirochaete, 1 member of the Fibrobacteres, 1 member of the Euryarchaeota and diverse members of the Proteobacteria. Thus Dissemination seems to capture more mechanistic rather than phylogenetic information, whereas heritage has more influence on the Sophistication score.

The metrics proposed here don't consider sequence similarity or orthology. relationships, and are particularly sensitive to gene gain/loss (Dissemination) and gene fusion/fission events (Sophistication). The non-requirement for sequence similarity data allows metrics to be calculated for genomes that are the sole examples within a clade, however superimposing an orthology analysis onto a metrics-based characterisation should provide information regarding the lability of orthologous systems, and how their gene/domain organisation has evolved.

Sophistication and Dissemination are direct measures of gene organisation, and are only indirectly affected by domain architecture. Metrics based on domain composition tended to be 'elitist', as the vast majority of TCS genes (~90%) contain only 1 domain, and are therefore of limited usefulness as discriminators. Metrics that avoid this problem by capturing (for example) the number of foci with >X domains/genes, correlate strongly with the total number of TCS genes, so are essentially modified versions of the IQ metric and of little novelty.

How then can the Dissemination and Sophistication scores be used? Beyond identifying replicons with unusually high or low scores, the next step is to identify factors that correlate with these two metrics. For instance in an earlier study it was found that the apparent fusion/fission rates of TCS genes correlated with the presence of TM helices and DNA-binding domains, and with gene order [16]. Similarly, analysis of the factors that co-vary with Dissemination and/or Sophistication should provide insights into the relationship between gene/focus structure and function (physiological and/or mechanistic).

## Conclusions

In this study we have used a rationally-devised scheme to classify gene and domain architectures of prokaryotic TCS genes and in doing so, identified general features of TCS organisation. Our analyses provide a baseline for comparison with individual organisms, and to such an end, we have also proposed two new metrics of TCS organisation that should prove useful in teasing apart features of TCS organisation and evolution.

## Authors' contributions

RHNW wrote all scripts, generated and analysed the datasets. DEW conceived of the study, directed the research and wrote the manuscript. Both authors read and approved the final manuscript.

## Supplementary Material

Additional file 1 **Numbers of TCS gene tetrads/triads/pairs/orphans per replicon**. For every replicon analysed, the total number of HK, PP and RR genes are presented, along with the numbers of TCS genes found as orphans, paired genes, triads and tetrads. In addition the types of TCS genes found as orphans, and in various paired-gene geometries are provided. Click here for file

Additional file 2 **Dataset of TCS gene foci domain/gene organisation**. A dataset of TCS gene foci, with each focus defined using the locus tag of the first gene in the focus. For each focus, gene architecture and domain organisation are presented. Click here for file
